# The New York School Medical Service

**Published:** 1912-10-26

**Authors:** 


### THE IDEAL SCHOOL CLINIC.

### I.—The New York School Medical Service.

### SOME INTERESTING COMPARISONS.

The New York Academy of Medicine some time ago appointed a special committee to investigate and report upon the existing school medical service in New York. This committee has now issued its final report, which gives a broad survey of the general scope of the system of medical inspection of schools in America, more particularly as it is in working in New York. Such a survey presents many points of obvious interest to school doctors on this side, and it is therefore permissible to deal with the report at some length.

In New York the study of the health conditions in the public schools has been carried on with five main objects in view. These are: (1) To summarise present methods of safeguarding the health of school-going children; (2) to analyse these methods and their results from a strictly medical point of view; (3) to enlist the co-operation of the entire medical profession in dealing with the problem of school hygiene; (4) to give medical advice to the Education Department; and, finally, (5) to help in maintaining proper health conditions in the schools. After dealing fully with the general objects of school medical inspection, the signatories of the report (among them being Drs. Dana, Bristow, Goldwater, Jacobi, and Le Fevre) point out that medical inspection was made compulsory in the Austrian schools as far back as 1873. In 1899 Russia came into line, in this respect, with all Continental nations except Germany. England followed in 1907. In Germany and in America such inspection is done locally and is not compulsory.

#### The Officials and Their Pay.

Yet so well have the municipalities in the United States realised their responsibilities that in 1911 no fewer than 1,038 cities had already appointed medical inspectors of schools. In New York, where the system has been planned on a large scale and where the organisation is perhaps more perfect than in any other city in the States, there were, in 1911, 126 doctors (each paid £250 per annum) and 134 nurses (each paid £180 per annum). This year (1912) only 76 doctors were engaged (at the same salary), but the staff of nurses was increased to 172 (also paid at the same rate—*i.e.* £180); while, in addition, six doctors were engaged (at a salary of £250) to supervise the work, four doctors (also at £250) to examine special children, and ten extra nurses (at £180) to act as district supervisors. It is claimed that by this reorganisation a saving of £800 was effected, while more efficient work was secured. Under the New York system the doctor works only four hours a day; he visits each school in his district two days in succession after intervals of ten days. At the head of the department is a director, an assistant director, and five borough chiefs, who give their whole time to the duties of school inspection. The other doctors are part-time officials, who have each some 8,000 to 10,000 children under their charge.

The school doctor on routine visits inspects from twenty to twenty-two children during the day. This, one notices at once, compares very favourably with the average of twenty-five required in London by the London County Council regulations. Consequently, the examinations are much more thorough. Whether that is altogether a good thing remains to be seen. Certainly the figures for defectives found strike one as being altogether out of all proportion to the reality. For example, in 1911, out of 230,243 children examined, no fewer than 166,368 (a percentage of 72.2) were noted as "needing medical treatment." In 1909 the percentage was even higher, 74.4. And these, we are specifically told, do not include "children reported with defective teeth as the only defect, whose treatment consisted only in instruction in oral hygiene."

#### National Comparisons.

We find it is interesting to note, when comparing the percentages of defects, that there is a wide diversity between certain defects, and that the figures of New York do not in the least correspond with those of London. For instance, defective vision accounts for only 10 per cent.: in London it is, perhaps, the most common defect noted. "Hypertrophied tonsils" (which may or may not be a defect) accounts for 15 per cent.; defective nasal breathing (a much better heading) for 11 per cent.; defective nutrition 2.5 per cent.; while cardiac cases and pulmonary disease have the relatively low percentages of .7 and .2 respectively. Orthopædic defects, we are astonished to find, are given as low as .5 per cent.; chorea as .3; tuberculous lymph nodes .2. Out of a total of 674,667 children, 286,591, or 42 per cent., were found to be suffering from communicable eye and skin diseases in 1910. More than half of these were cases of pediculosis, while 16 per cent. were cases of trachoma. Indeed the figures given in the report in regard to communicable diseases are most interesting and clearly show that the systematic inspection of school children has an undoubtedly high hygienic value. In 1909, 286,591 such cases were excluded; in 1911 the number was 248,771, but the percentage of the more serious conditions had fallen to less than a quarter. Thus, in 1909, 45,615 trachoma cases were excluded; in 1911 only 15,245; in 1909 4,006 cases of scabies and 409 of favus were excluded; in 1911 the figures for these diseases were 1,768 and 220 respectively. The figures with regard to major contagious diseases are, as is to be expected, less satisfactory. In 1911 some 1,595 (1,340 true diagnoses) cases were excluded; in 1912 1,707 (1,159 true diagnoses) were similarly excluded.

Both nurses and doctors are allowed to exclude children on diagnosing such diseases as scarlet fever, measles, diphtheria, mumps, tuberculosis, whooping-cough, and chicken-pox. It is interesting to find that the nurses' diagnoses are much more fallacious than the doctors': in scarlet fever 63.3 per cent. of nurses' exclusions were true cases, while 66.6 per cent. of doctors' cases were true; in measles 56 per cent. of nurses' and 85 per cent. of doctors' cases were true; in mumps 78 per cent. nurses' and 100 per cent. doctors' cases were true. The nurses err on the side of caution and exclude all suspected cases.

This dual control in regard to excluding suspect cases cannot be expected to work well, and it is not to be wondered at that the committee reports against it. The committee remarks: "Reports are unanimous in disapproving of nurses diagnosing and excluding contagious cases, for the following reasons: Their training has not fitted them for it, physically or mentally. They are overworked, and unable, for lack of time, to perform their other duties, especially home visits, which is the essential part of their work. There is a duplication of work, because inspectors have to visit each case excluded. The nurses exclude many false cases, thereby causing the inspectors to waste much time in making unnecessary visits. The medical inspector is not able to keep in close touch with the school on account of the infrequency of his visits, so the school physician no longer exists, and the nurse cannot take his place. Principals and parents naturally do not have the same confidence in her judgment as they have in the physician's."

### Lessons from New York.

Under the New York system each child is examined upon entering and leaving school, and in addition every two and a half years, on an average, in the interim. Yet, although the number of defective children found is apparently so large that one is inclined to believe that the New York school children belong to a physically deteriorating race (which we for our part refuse to believe), and although the school doctor has ample time for his examination, the inspections are not so thorough as one may at first imagine. Attention is directed chiefly to the more or less chronic physical defects of the eye, ear, nose and throat, heart, lungs and joints. Yet this examination is done without divesting the child of its clothing! "One of the reasons for this given by the New York Department of Health," we are told, "is that parents would object to having examinations of girls made by school physicians." The report therefore agrees that the examinations are not thorough, and the committee recommends (recommendation 4) as follows:—"Physical examinations should be made more thorough and more frequent. The children, or at least the boys at first, should be stripped to the waist at physical examinations. The present plan of examining the child when it enters the school, when it graduates (*i.e.*, leaves school), and once in the interim should be changed. A child should be examined when it enters school and then every two years. The examination before leaving school does not have any particular importance." With this we thoroughly agree. If a child, in a particular case, needs examination before leaving, in order to find out whether it is physically competent to undertake work of a certain nature, it can be examined as a special case. Usually in such a case the school doctor, at his routine inspections, will have noted whatever anomaly is present and instructed parents and teachers accordingly.

The New York report shows very clearly that it is useless to expect uniformity in an organisation which depends largely on half-time officials. In their examinations the personal equation so largely enters that it is impossible even to find a means adequately to correlate statistics gathered from routine inspections.

### Discrepancies in the Findings.

"In 1908 the Bureau of Municipal Research conducted an inquiry into the methods and results of the examinations. It was found then that the inspectors differed widely in the number and kinds of defects found in the same children examined. Discrepancies as great as between 30 per cent. and 92 per cent. and between 43 per cent. and 84 per cent. occurred in the same schools, while the variation in percentages found defective in the whole of Manhattan was from 100 per cent. to 32 per cent., and from 100 per cent. to 18 per cent. in Brooklyn" (Cf. Report of Bureau of Municipal Research. Child Hygiene. 1908. Pp. 11-14). Under the part-time system of the London County Council similar discrepancies occurred, and even now, when the part-time staff has been displaced almost wholly by whole-time officials, there is still a lamentable lack of uniformity in putting down defects.

This is due to the different interpretation of what constitutes a defect by different examiners. Thus, one doctor will only consider hypertrophied tonsils a defect when they need treatment; another will consider them a defect in any case. Again, an error of vision not exceeding $\frac{1}{1}\frac{2}{2}$ in a child in the infants' department will be considered by some as a defect not requiring treatment, whereas another will report it as a defect needing treatment. There is need, therefore, for a concise and clear and authoritative model which should be followed by all examiners. We do not think the Memorandum drawn up by the Board of Education supplies that want, and we should like to see the Association of Medical Officers of Schools appoint a sub-committee to investigate the matter and deal with it as soon as possible. Without uniformity in regard to the interpretation of findings, the results of examinations, so far as statistical value is concerned, will be next to nothing. With uniformity it may amount to much, for it cannot reasonably be doubted that these routine inspections of normal children will in course of time supply us with very instructive data.

In the following article we hope to deal with the important point of treatment and the various recommendations contained in the New York report.

